# Discovery of an antibody for pan-ebolavirus therapy

**DOI:** 10.1038/srep20514

**Published:** 2016-02-10

**Authors:** Wakako Furuyama, Andrea Marzi, Asuka Nanbo, Elaine Haddock, Junki Maruyama, Hiroko Miyamoto, Manabu Igarashi, Reiko Yoshida, Osamu Noyori, Heinz Feldmann, Ayato Takada

**Affiliations:** 1 Division of Global Epidemiology, Research Center for Zoonosis Control, Hokkaido University, Sapporo, Japan; 2 Laboratory of Virology, Division of Intramural Research, National Institute of Allergy and Infectious Diseases, National Institutes of Health, Rocky Mountain Laboratories, Hamilton, Montana, USA; 3 Department of Cell Physiology, Graduate School of Medicine, Hokkaido University, Sapporo, Japan

## Abstract

During the latest outbreak of Ebola virus disease in West Africa, monoclonal antibody therapy (e.g., ZMapp) was utilized to treat patients. However, due to the antigenic differences among the five ebolavirus species, the current therapeutic monoclonal antibodies are only effective against viruses of the species *Zaire ebolavirus*. Although this particular species has indeed caused the majority of human infections in Central and, recently, West Africa, other ebolavirus species (e.g., *Sudan ebolavirus* and *Bundibugyo ebolavirus*) have also repeatedly caused outbreaks in Central Africa and thus should not be neglected in the development of countermeasures against ebolaviruses. Here we report the generation of an ebolavirus glycoprotein-specific monoclonal antibody that effectively inhibits cellular entry of representative isolates of all known ebolavirus species *in vitro* and show its protective efficacy in mouse models of ebolavirus infections. This novel neutralizing monoclonal antibody targets a highly conserved internal fusion loop in the glycoprotein molecule and prevents membrane fusion of the viral envelope with cellular membranes. The discovery of this highly cross-neutralizing antibody provides a promising option for broad-acting ebolavirus antibody therapy and will accelerate the design of improved vaccines that can selectively elicit cross-neutralizing antibodies against multiple species of ebolaviruses.

Ebolaviruses, members of the family *Filoviridae*, cause severe hemorrhagic fever in humans and nonhuman primates, with human case fatality rates of up to 90%^1^,^2^. As proven by the latest epidemic of Ebola virus disease (EVD) in West Africa, ebolaviruses pose a significant public health concern. However, no effective prophylaxis or treatment for EVD is as yet commercially available. Five distinct species are known in the genus *Ebolavirus, Zaire ebolavirus, Sudan ebolavirus, Taï forest ebolavirus, Bundibugyo ebolavirus*, and *Reston ebolavirus*, represented by Ebola virus (EBOV), Sudan virus (SUDV), Taï forest virus (TAFV), Bundibugyo virus (BDBV), and Reston virus (RESTV), respectively^3^. Of these, EBOV, SUDV, and BDBV have caused EVD outbreaks with increased frequency in Central and West Africa in the last decade^1^,^2^,^4^.

Ebolaviruses express a single transmembrane glycoprotein (GP) that is responsible for both receptor binding and membrane fusion, and thus the only known target of neutralizing antibodies. GP undergoes proteolytic cleavage by host proteases such as furin, resulting in the two subunits, GP1 and GP2, which are linked by a disulfide bond^5^,^6^. The GP1 subunit (amino acids 33–501) contains the core of the glycoprotein, receptor binding domain (RBD), a glycan cap, and a large mucin-like domain which extends around the RBD^7^. The GP2 (amino acids 502–676) subunit contains the internal fusion loop (IFL), heptad repeats 1 and 2 (HR1 and HR2), the transmembrane region (TM), and the cytoplasmic tail (CT)^8^. During the transport of ebolavirus particles to late endosomes, low pH leads to proteolytic processing of GPs by host cysteine proteases such as cathepsins^9^, and the exposed receptor binding site of the proteolytically digested GP is thought to interact with a host receptor, Niemann Pick C1, followed by membrane fusion^8^,^10^,^11^.

Several studies have demonstrated that administration of EBOV GP-specific antibodies protects nonhuman primates from lethal EBOV infection and may constitute a leading treatment option for EVD in humans^12^,^13^,^14^,^15^,^16^. During the West African EVD outbreak, EBOV GP-specific monoclonal antibody (MAb) cocktails (e.g., ZMapp)^12^, were used to treat several patients^17^,^18^,^19^,^20^. However, most of characterized therapeutic MAbs to date are EBOV GP-specific and cross-neutralizing activity against any other ebolavirus species (e.g., SUDV and BDBV) has not been demonstrated due to antigenic differences among the species^21^. Since SUDV and BDBV have also shown their potential to cause public health emergencies during several outbreaks in Central Africa, it is difficult to determine the priority for development of countermeasure against those ebolaviruses.

Here we report a broadly cross-reactive GP-specific MAb. This MAb, 6D6, recognizes the putative epitope in the highly conserved IFL and neutralizes infectivity of representative isolates of all known ebolavirus species by inhibiting the membrane fusion. We further demonstrate its protective potential as a therapeutic antibody in mouse models of EBOV and SUDV infections.

## Results

### *In vitro* properties of MAb 6D6

**Figure 1:**
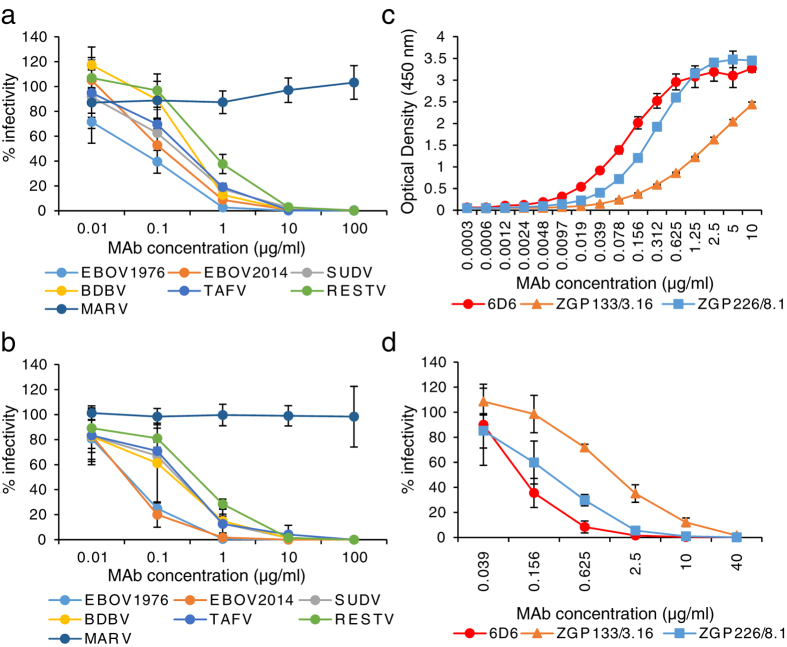
Neutralizing properties of MAb 6D6 against ebolaviruses. (**a**) VSV pseudotyped with the indicated GPs or (**b**) infectious EBOV, SUDV, TAFV, BDBV, RESTV, and MARV were incubated with purified MAb 6D6 followed by inoculation into confluent Vero E6 cells. (**c**) Binding activities of MAbs 6D6 (red), ZGP133/3.16 (orange) and ZGP226/8.1 (blue) were examined by ELISA using EBOV GP as the antigen. (**d**) Neutralizing activities of MAbs 6D6 (red), ZGP133/3.16 (orange), and ZGP226/8.1 (blue) against VSV pseudotyped with EBOV GP are shown. The mean and standard deviation of three independent experiments are shown.

The cross-reactive MAb (6D6) was selected by screening mouse hybridoma supernatants thoroughly for the cross-neutralizing activity of GP-specific MAbs. MAb 6D6 was found to be GP-specific and to efficiently neutralize the infectivity of vesicular stomatitis virus (VSV) pseudotyped with GPs of all known ebolaviruses (EBOV, SUDV, TAFV, BDBV, and RESTV), including the variant that caused the latest outbreak in West Africa (EBOV2014), but not Marburg virus (MARV), a related filovirus that causes human disease similar to EVD (Fig. 1a). The 50% inhibitory concentrations (IC_50_) of 6D6 for VSVs bearing EBOV1976, EBOV2014, SUDV, TAFV, BDBV, and RESTV GPs were 0.05, 0.12, 0.19, 0.33, 0.24, and 0.62 μg/ml, respectively. We then confirmed that 6D6 effectively neutralized the infectivity of representative authentic isolates of all known ebolavirus species (Fig. 1b). Furthermore, binding experiments to EBOV GP and neutralization assays with EBOV GP-pseudotyped VSV revealed that 6D6 possessed higher binding and neutralizing abilities than EBOV GP-specific MAbs ZGP133/3.16 and ZGP226/8.1 (Fig. 1c,d), which have shown promising protective efficacy in animal models of lethal EBOV infection^14^,^22^.

### Identification of the putative 6D6 epitope

**Figure 2:**
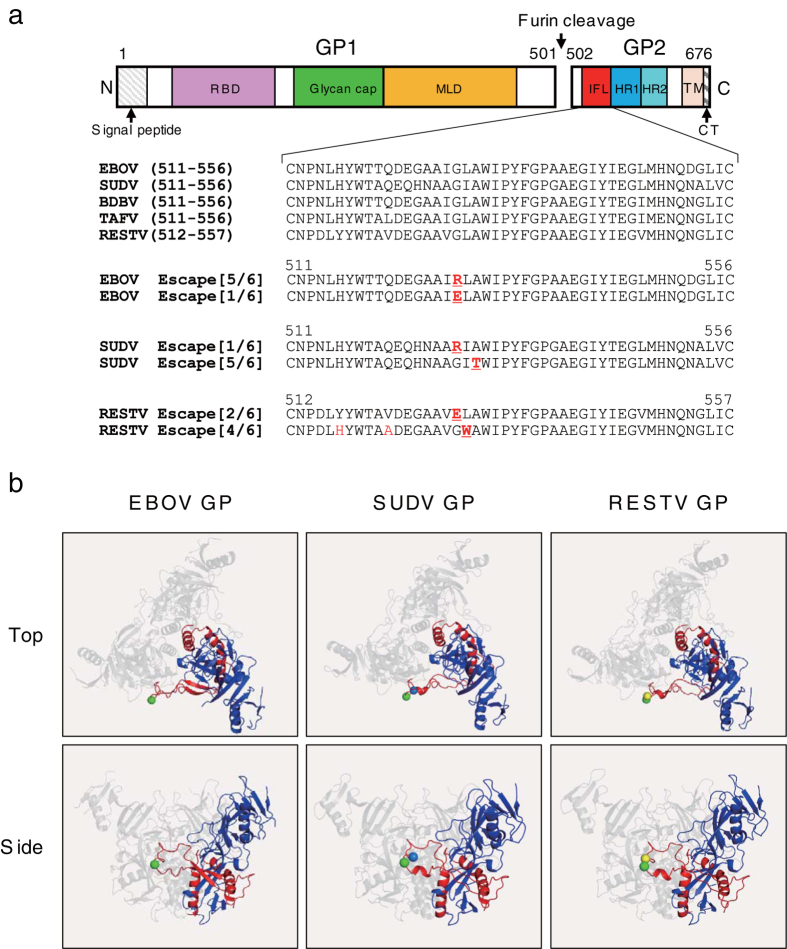
Identification of the putative epitope of MAb 6D6. (**a**) Structure of GP and amino acid sequences of the internal fusion loop (IFL). The GP1 subunit contains the receptor binding domain (RBD), a glycan cap and a mucin-like domain (MLD). The GP2 subunit contains the IFL, heptad repeats 1 and 2 (HR1 and HR2), the transmembrane region (TM), and the cytoplasmic tail (CT). Amino acid substitutions found in the EBOV, SUDV, and RESTV GP escape mutants selected under MAb 6D6 pressure are shown in red. (**b**) The amino acid residues (green, blue, and yellow spheres represent Gly, Ala, and Leu, respectively) critical for escape from the MAb 6D6 neutralization are mapped on the trimeric structure of GPs. GP1 (blue) and GP2 (red) monomers are shown as ribbon models.

To determine the putative epitope of MAb 6D6, we utilized replication-competent recombinant VSV containing the EBOV, SUDV, or RESTV GP gene^23^. The putative epitopes of ZGP133/3.16 and ZGP226/8.1 have been successfully determined by identifying the amino acid substitutions observed in the antigenic variants escaping from neutralization by the antibodies^23^,^24^. We cloned 6 escape mutants of EBOV GP and found that each mutant had a single amino acid substitution, Gly-to-Arg (5/6) or Gly-to-Glu (1/6), at amino acid position 528 within the IFL sequence in the GP2 subunit (Fig. 2a). One of the six SUDV GP escape mutants had a Gly-to-Arg substitution at position 528, and other 5 SUDV GP escape mutants had an Ala-to-Thr substitution at position 530 (Fig. 2a). Two of the six RESTV GP escape mutants had a Gly-to-Glu substitution at position 529, which corresponded to position 528 of EBOV GP. A total of 3 amino acid changes were found in the other 4 RESTV GP escape mutants (Fig. 2a). Using a reverse genetics approach we verified that the Leu-to-Trp substitution at position 530 was critical for escape from 6D6 neutralization (Supplementary Fig. 1). These amino acid positions, which are located at the tip of the IFL structures of EBOV, SUDV, and RESTV GPs, indicate that the loop structure including these residues is important to form the recognition site of 6D6 (Fig. 2b). We confirmed that 6D6 did not bind to the chimeric EBOV GP whose IFL region was replaced with that of MARV; however, 6D6 showed no binding activity to the synthetic peptide corresponding to the amino acids of the IFL of EBOV GP (not shown), suggesting that the 6D6 epitope may partly include other conformational structures. Importantly, the amino acid sequence of the IFL region is highly conserved among all currently known ebolaviruses (Fig. 2a), providing a novel target for universal antibody therapy against EVD caused by human-pathogenic ebolaviruses (EBOV, SUDV, TAFV, and BDBV).

### Mechanism of the neutralizing activity of 6D6

**Figure 3:**
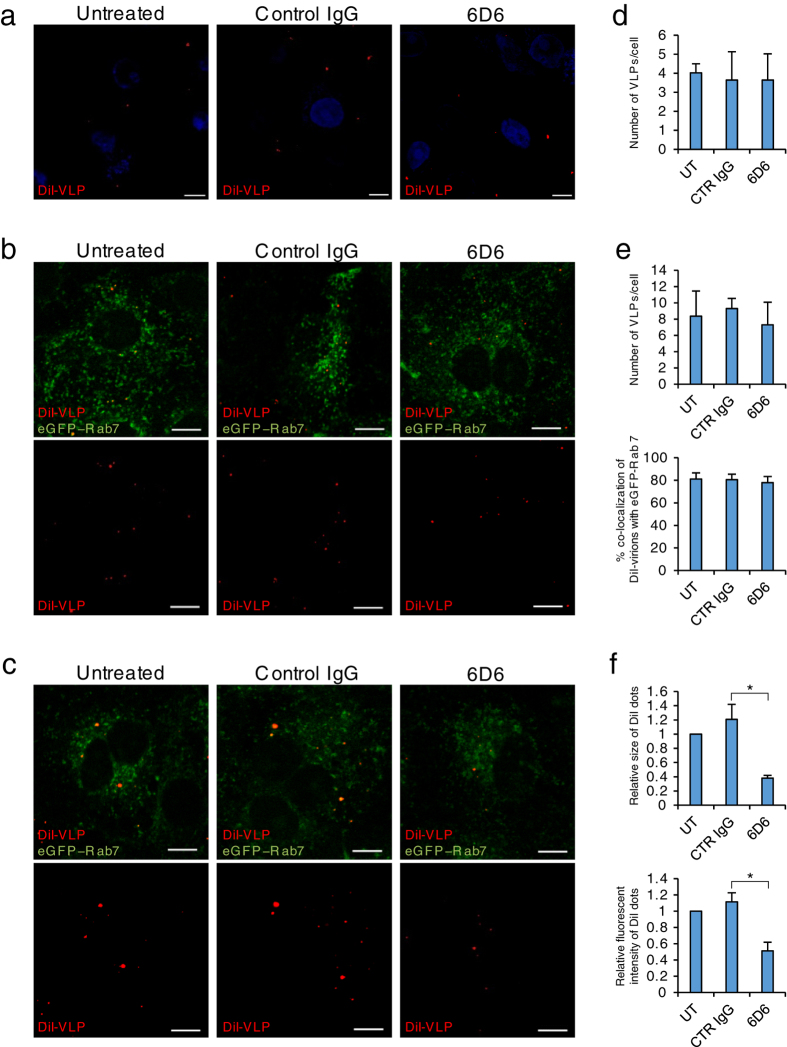
Effect of MAb 6D6 on virus entry. (**a–f**) Untreated (UT), control IgG (CTR IgG)- or 6D6-treated DiI-labelled VLPs were inoculated into confluent Vero E6 cells expressing eGFP-Rab7 and incubated for 30 min on ice. After adsorption, the cells were incubated for 0 (**a**,**d**), 2 (**b**,**e**) and 6 h (**c,f**) at 37 °C. (**a–c**) DiI signals on the cell surface (**a**) and in the cytoplasm (**b**,**c**) were monitored by confocal laser scanning microscopy. (**d–f**) The number, size, and fluorescence intensity of DiI dots were quantified. Scale bars represent 10 μm. The means and standard deviations of three independent experiments are shown. Statistical analysis was performed using a Student’s *t*-test (*p < 0.05).

Since the IFL is crucial for GP-mediated membrane fusion, we assumed that 6D6 directly inhibited the fusion step during the entry process of ebolaviruses into cells. To confirm this, we analyzed the inhibitory effects of 6D6 on viral attachment, internalization, and membrane fusion using lipophilic tracer (DiI)-labelled virus-like particles (VLPs)^25^. The number of 6D6-treated VLPs attached to the surface of Vero E6 cells was not significantly different from that of untreated or control IgG-treated VLPs, indicating that 6D6 did not interfere with VLP attachment (Fig. 3a,d and Supplementary Fig. 2). Likewise, the number of VLPs that colocalized with eGFP-Rab7, a late endosome marker, was similar, suggesting that 6D6 did not affect subsequent uptake into cells (Fig. 3b,e and Supplementary Fig. 2). Finally, we analyzed membrane fusion efficiency by detecting dequenched DiI fluorescence^25^,^26^. We observed remarkably enlarged and enhanced DiI signals colocalizing with Rab7 in cells incubated with untreated and control IgG-treated VLPs, indicating that membrane fusion occurred efficiently in the endosomes (Fig. 3c left and middle panels). In contrast, the size and intensity of DiI signals from 6D6-treated VLPs were significantly reduced, indicating that 6D6 prevented GP-mediated membrane fusion (Fig. 3c right panels, f and Supplementary Fig. 2).

### Protective efficacy of MAb 6D6 in mouse models

**Figure 4:**
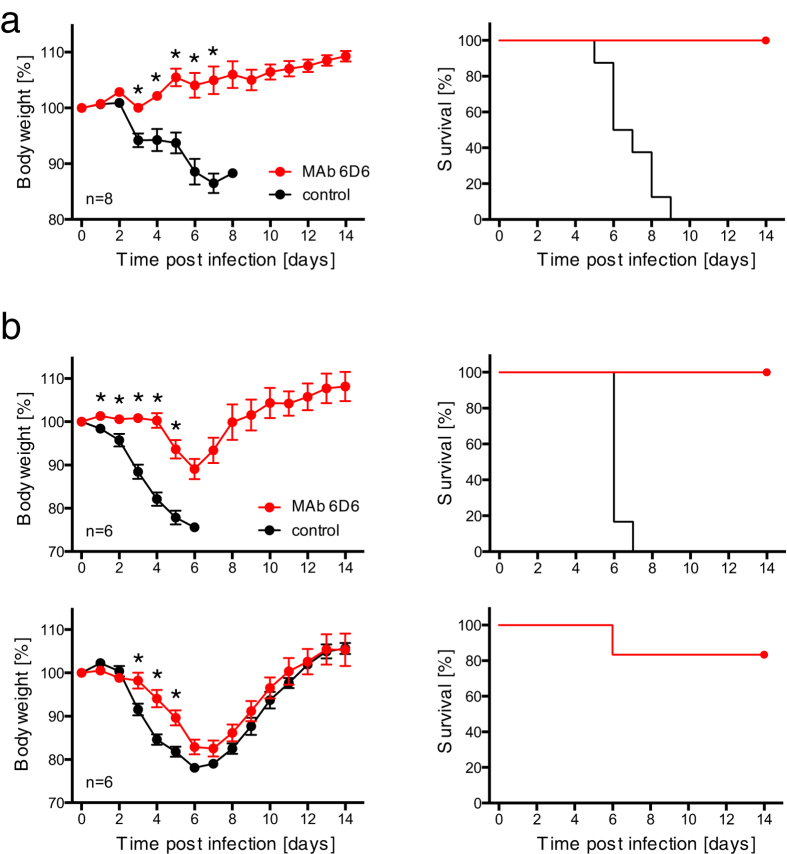
Protective efficacy of MAb 6D6 in mice. (**a**) BALB/c mice (n = 8) were intraperitoneally infected with a lethal dose of mouse-adapted EBOV. (**b**) IFNAR^−/−^ mice (n = 6) were intraperitoneally infected with EBOV1976 (upper panels) or SUDV (bottom panels). Twenty-four hours after infection, animals were treated intraperitoneally with either 100 μg of MAb 6D6 or a vehicle control (PBS). The animals were then monitored for 14 days for clinical signs of infection and weighed daily. Body weight (left panels) and survival curves (right panels) are shown. Error bars indicate standard error of the mean. Significant differences are indicated by asterisks (*p < 0.05).

Finally, we investigated the potential of 6D6 to protect mice from ebolavirus infections (Fig. 4). Immunocompetent BALB/c mice were infected with a lethal dose of mouse-adapted EBOV and treated 24 h later with 100 μg of 6D6. The treated animals survived without clinical symptoms, whereas untreated mice succumbed to infection within 9 days (Fig. 4a). We further evaluated the cross-protective potential of 6D6 against wild-type EBOV and SUDV infections (Fig. 4b). Since immunocompetent mice do not develop disease upon infection with these wild-type ebolavirus isolates, interferon α/β receptor knockout (IFNAR^−/−^) C57BL/6 mice were used for this purpose. We found that both EBOV and SUDV caused severe weight loss in untreated mice, whereas only EBOV uniformly caused a lethal infection. Treatment with 6D6 24 h after infection delayed the onset of the disease caused by these ebolaviruses and significantly reduced the weight loss in this immunocompromised mouse strain. All 6D6-treated mice survived the EBOV infection.

## Discussion

Neutralizing antibody-based therapies have been tested in animal models and clinical trials with particular attention given to highly lethal viral diseases^27^. Passive immunization with convalescent serum has also been tested for EBOV-infected patients^28^,^29^. Recent studies have demonstrated the effectiveness of MAb treatments in nonhuman primate models of EVD^12^,^14^,^15^,^16^,^30^,^31^, and GP-specific MAb cocktails, ZMapp and ZMAb, were used in clinical cases during the 2014 EVD outbreak caused by EBOV (belonging to *Zaire ebolavirus*)^17^,^18^,^19^,^20^. However, these MAb cocktails are not expected to be cross-protective against the other antigenically distinct ebolaviruses. On the other hand, highly cross-reactive MAbs against all known ebolaviruses have been generated previously, but none of those has neutralizing activity^21^,^32^. In this study, we generated the novel MAb 6D6, which has cross-neutralizing activity against all known ebolaviruses.

We showed that 6D6 reduced infectivities of all known ebolaviruses *in vitro* with higher neutralizing and binding activities than the EBOV GP-specific MAbs ZGP133/3.16 and ZGP226/8.1. Indeed, the IC_50_ values of 6D6 were equal to or lower than those of previously reported neutralizing MAbs^33^,^34^,^35^, suggesting its protective capacity as a therapuetic MAb. By analyzing the amino acid substitution observed in the antigenic variants escaping from 6D6, we determined the putative epitope of 6D6 in the IFL on the GP molecule, which may overlap that of a partially cross-reactive GP-specific MAb reported recently^36^. Accordingly, 6D6 directly inhibited the membrane fusion induced by EBOV VLPs in endosomes/lysosomes. The IFL structure is highly conserved in all species of ebolaviruses, indicating that this region can be targeted for both vaccine and therapeutic development against ebolaviruses. Since the putative epitope of 6D6 is different from those previously reported for other GP-specific MAbs used in passive immunization studies^23^,^34^,^37^,^38^,^39^,^40^, 6D6 may provide a promising option as a component of antibody cocktails in combination with other previously tested MAbs.

We further demonstrated that passive immunization with 6D6 protected both BALB/c and IFNAR^−/−^ C57BL/6 mice from lethal infection by EBOV (i.e., mouse-adapted and wild-type EBOV, respectively). However, the cross-protective potential of 6D6 could only be evaluated for disease severity using IFNAR^−/−^ C57BL/6 mice since mouse-adapted SUDV causing lethal infection in immunocompetent mice is not currently available and SUDV did not uniformly cause lethal infection even in this immunocompromised mouse strain. We found that passive immunization with 6D6 significantly reduced the weight loss in SUDV-infected mice, although the extent was not as prominent as in EBOV-infected mice. The less significant protective effects seen in SUDV-infected mice might have been due to the higher IC_50_ value of 6D6 against SUDV than against EBOV. Thus, these results supported the *in vitro* characteristics of 6D6 and demonstrated the effectiveness of the 6D6 treatment *in vivo* against multiple species of ebolaviruses. A previous study demonstrated that immunocompromised mice treated several times with 500 μg of MAb SUDV-specific MAbs were protected from SUDV infection^41^, whereas mice were treated once with 100 μg of 6D6 in this study. Thus, we assume that the protective effect against SUDV could be improved by increased doses of 6D6.

The broadly cross-neutralizing antibody 6D6 recognizing the common epitope shared among all currently known ebolaviruses, converted into a human-mouse chimeric MAb^14^, is a promising therapeutic candidate. On the other hand, the generation of 6D6 escape mutants *in vitro* speaks against monotherapy with this MAb and may favor the development of antibody cocktails including 6D6 for future pan-ebolavirus therapy. For other viruses, it has indeed been reported that combination of MAbs helps to avoid the appearance of escape variants if these MAbs recognize distinct epitopes^42^,^43^,^44^. While the detailed mechanisms underlying antibody-mediated protection from ebolavirus infection need to be further elucidated, the discovery of this highly cross-reactive neutralizing antibody and its putative epitope reported here provides a promising option for the development of a universal EVD therapy and will accelerate the design and implementation of improved therapeutics and vaccines that can selectively elicit cross-neutralizing antibodies against multiple species of ebolaviruses.

## Methods

### Viruses and cells

Variants Yambuku (EBOV1976), Makona (EBOV2014), Nzara (SUDV), Butalya (BDBV), Pauléoula (TAFV), Philippines89 (RESTV) and Angola (MARV), and mouse-adapted EBOV^45^ were propagated in Vero E6 cells and stored at −80 °C. Virus titers were determined as focus forming units (FFU) by immunoplaque assays. All infectious work with filoviruses was performed in the biosafety level 4 laboratories at the Integrated Research Facility of the Rocky Mountain Laboratories, Division of Intramural Research, National Institute of Allergy and Infectious Diseases, National Institutes of Health, Hamilton, Montana, USA. Replication-competent recombinant VSV (rVSV/EBOV GP, rVSV/SUDV GP, and rVSV/RESTV GP) and replication-incompetent pseudotyped VSVs containing GFP instead of the VSV G gene were generated as described previously^23^,^46^. Infectious units (IUs) of replication-incompetent pseudotyped VSVs were determined using Vero E6 cells as described previously^46^. African green monkey kidney Vero E6 cells and human embryonic kidney (HEK) 293T cells were grown in Dulbecco’s modified Eagle’s medium (DMEM) (Sigma). The media were supplemented with fetal calf serum (FCS) (Cell Culture Bioscience) and 100 U/ml penicillin, 0.1 mg/ml streptomycin (Gibco).

### Purification of VLPs for immunization

HEK293T cells were transfected with equal amounts of the expression plasmids encoding GP, matrix protein (VP40), and nucleoprotein (NP) of EBOV or SUDV using TransIT LT-1 reagent (Mirus) according to the manufacturer’s instructions. Forty-eight hours later, the culture supernatant was harvested and centrifuged at 3,500 rpm for 15 min to remove cell debris. VLPs were purified from culture supernatants by ultracentrifugation at 28,000 rpm with an SW32Ti rotor (Beckman) at 4 °C for 2 h with a 25% sucrose cushion. The VLP pellets were suspended in phosphate-buffered saline (PBS) and fractionated through a 20–50% sucrose gradient in PBS at 28,000 rpm with an SW41 rotor (Beckman) at 4 °C for 2 h. Then the VLP fractions were diluted with PBS and sedimented by ultracentrifugation at 28,000 rpm with an SW41 rotor at 4 °C for 2 h. Finally, the VLP pellets were resuspended in PBS.

### Generation of MAb 6D6

Fifteen-week-old female BALB/c mice were immunized intraperitoneally with 100 μg of EBOV VLPs. At 2 and 5 weeks after the first immunization, the mice were intraperitoneally immunized with 100 μg of EBOV VLPs. At 10 weeks after the first immunization, the mice were immunized with 100 μg of SUDV VLPs. Two weeks after the last immunization, the mice were boosted intraperitoneally with 100 μg of EBOV VLPs. Three days later, the mice were euthanized and spleen cells and mouse myeloma P3U1 cells were fused and maintained according to a standard procedure^47^. The mice were treated daily with 75 μg/kg rapamycin intraperitoneally starting 1 week prior to the primary immunization until euthanasia. Hybridomas were screened for secretion of EBOV GP-specific MAbs by a neutralization test with VSV pseudotyped with EBOV GP and hybridomas producing MAbs were cloned twice by limiting dilution of the cells. Hybridomas producing neutralizing MAbs were further screened for the cross-reactivity to the other filovirus GPs. MAb 6D6 (IgG1) was found to be a broadly cross-neutralizing MAb and was purified from mouse ascites using protein A agarose columns (Bio-Rad). Animal studies were carried out in strict accordance with the Guidelines for Proper Conduct of Animal Experiments of the Science Council of Japan. The protocol was approved (13–0136) by the Hokkaido University Animal Care and Use Committee.

### Neutralization tests

To evaluate the neutralizing activity a focus forming assay was used. Dilutions of EBOV, SUDV, TAFV, BDBV, RESTV, and MARV (20–100 FFU/0.1 ml) were mixed with purified MAb 6D6 for 1 h at 37 °C and inoculated into confluent Vero E6 cells grown in 96-well tissue culture plates. After incubation for 3 days, the cells infected with filoviruses were fixed and stained with a mixture of anti-GP (anti-EBOV 42/3.7 or anti-MARV FS0505) and anti-NP (anti-EBOV 74/7 or anti-MARV FS0609) primary antibodies^26^ followed by anti-mouse IgG/Alexa Fluor 488 (A11029, Invitrogen) and anti-rabbit IgG/Alexa Fluor 488 (A11034, Invitrogen) secondary antibodies. Virus infectivity was quantified by counting the number of fluorescent foci. VSV pseudotyped with filovirus GPs were appropriately diluted to yield 300 to 1,500 IUs and mixed with purified MAb 6D6, ZGP133/3.16, or ZGP226/8.1 for 1 h at room temperature, and inoculated into confluent Vero E6 cells grown in 96-well plates. At 20 h postinoculation, GFP-positive cells were counted using IN Cell Analyzer 2000 (GE Healthcare). To reduce the background infectivity of the parent VSV G, pseudotyped viruses were treated with a neutralizing MAb to VSV G protein (VSV-G[N]1-9) before use. The relative percentage of infectivity was calculated by setting the number of cells infected in the absence of MAb 6D6 to 100%.

### Enzyme-linked immunosorbent assay (ELISA)

GP-based ELISA was performed as described previously^21^. Soluble forms of EBOV GP were purified and used as antigens. MAbs were serially diluted with PBS containing 0.05% Tween 20 and 1% skim milk. Bound antibodies were visualized with horseradish peroxidase-conjugated goat anti-mouse IgG (H + L) (Jackson ImmunoResearch) and 3,3′,5,5′-tetramethylbenzidine (Sigma). The reaction was stopped by adding 1 N phosphoric acid, and the optical density at 450 nm (OD_450_) was measured using SoftMax® Pro 6.2.1 software (Molecular Devices).

### Selection of escape mutants and identification of the putative epitope

Tenfold serial dilutions of rVSV/EBOV GP, rVSV/SUDV GP, and rVSV/RESTV GP were incubated with 10 μg/ml MAb 6D6 for 1 h at room temperature and inoculated into confluent Vero E6 cells grown in 6-well tissue culture plates. After adsorption for 1 h, the cells were overlaid with Eagle’s minimal essential medium (Invitrogen) containing 0.8% Bacto Agar (BD), 0.3% bovine serum albumin (Sigma), 100 U/ml penicillin, 0.1 mg/ml streptomycin, and 10 μg/ml 6D6, and then incubated for 2 days at 37 °C. Mutant viruses growing in the presence of MAb 6D6 were purified from single isolated plaques at the highest dilution of the virus and propagated in Vero E6 cells. Viral RNAs were extracted from the supernatant, the nucleotide sequences of the GP genes of the parent viruses and the escape mutants were determined and the deduced amino acid sequences were compared among these viruses. The IFL amino acid sequences of EBOV, SUDV, BDBV, TAFV and RESTV were obtained from GenBank (Accession numbers, U23187.1, U28134.1, NC_014373.1, U28006.1 and AF522874.1, respectively). The substituted amino acid positions were mapped on the trimeric structure of GPs constructed using Discovery Studio 4.1 (Biovia) based on the crystal structure of EBOV GP (PDB code: 3CSY). The SUDV and RESTV GP structures were generated by homology modelling based on the EBOV GP structure. From 100 models of the GP trimer, the model with the best score for probability density function (PDF) and total energy was chosen. The model was evaluated using Profiles-3D^48^.

### Purification and fluorescent-labelling of VLPs

For purification of VLPs, equal amounts of the expression plasmids for EBOV VP40, NP, and GP were transfected into HEK293T cells by using TransIT LT-1 (Mirus). Forty-eight hours post-transfection, the culture supernatant was harvested and centrifuged at 3,500 rpm for 15 min to remove cell debris. VLPs were precipitated through a 25% sucrose cushion by centrifugation at 11,000 rpm for 1 h at 4 °C with an SW32Ti rotor (Beckman). Precipitated VLPs were suspended in PBS, and fractionated through a 20–50% sucrose gradient in PBS at 27,000 rpm with an SW41 rotor (Beckman) for 2.5 h at 4 °C. One ml of fractionated VLPs (1 μg/ml) was incubated with 0.6 μl of 100 μM stock solution of 1,1′-dioctadecyl-3,3,3′,3′-tetramethylindocarbocyanine perchlorate (DiI) (Invitrogen) in the dark for 1 h at room temperature with gentle agitation^25^.

### Imaging of attachment, internalization and membrane fusion of DiI-labelled VLPs in live cells

Vero E6 cells expressing eGFP-Rab7 were cultured in 35 mm glass-bottom culture dishes (MatTek Corporation). DiI-labelled VLPs were treated with 20 μg/ml 6D6 or control IgG (mouse IgG1,κ; BD Biosciences) for 1 h at room temperature. The cells were then washed with 1 ml of phenol red-free DMEM (Invitrogen) and incubated with either MAb 6D6-treated, control IgG-treated or untreated VLPs in the same medium on ice for 30 min. Following this, they were washed with the same medium to remove unbound VLPs and incubated with 200 μl of phenol red-free DMEM containing 2% FCS and 4% bovine serum albumin at 37 °C for 0, 2, and 6 h to analyze attachment, internalization and membrane fusion, respectively. In this assay, the fluorescent signal is enhanced once the DiI-labelled VLP envelope fuses with the endosomal membrane^25^. To count the number of DiI-labelled VLPs, the cells were fixed in 4% paraformaldehyde for 15 min at room temperature. Then nuclei were stained using 1 μg/ml of 4′,6-diamidino-2-phenylindole, dihydrochloride (DAPI) for 10 min at room temperature (Thermo Fisher Scientific). Images were acquired with a 63× oil objective lens on a Zeiss LSM700 inverted microscope and ZEN 2009 software (Carl Zeiss). For measurement of the number of DiI-labelled VLPs, images of 4–20 optical sections were acquired in 0.5–1 micron steps. The number of DiI signals was determined in approximately 100 individual cells (approximately 1–20 dots/cell) and the average number per cell was calculated for each condition. For colocalization analysis, the percentage of DiI-labelled VLPs that colocalized with eGFP-Rab7-positive vesicles was measured using the Coloc module in ZEN 2010 software (Carl Zeiss). The number, size, and fluorescence intensity of DiI dots were analyzed with MetaMorph software (Molecular Devices). The relative sizes and intensities of DiI dots were determined by defining the value of untreated cells as 1.

### Passive immunization and protective efficacy in mice

BALB/c mice (female, 6–8 weeks old) were inoculated with mouse-adapted EBOV (1,000 FFU) by intraperitoneal (i.p.) injection in a total volume of 200 μl. One day after infection, the mice were treated with 100 μg of MAb 6D6 i.p. in a volume of 200 μl. To investigate the potential of 6D6 to protect mice from EBOV and SUDV infection, C57BL/6 IFNAR^−/−^ mice were chosen for this study as they are known to be susceptible to EBOV and SUDV infection. BDBV did not cause clinical symptoms in IFNAR^−/−^ mice (data not shown). IFNAR^−/−^ mice (male and female, 5–8 weeks old) were treated with 100 μg of MAb 6D6 i.p. in a volume of 200 μl one day after infection with EBOV1976 (1,000 FFU) or SUDV (1,000 FFU). The animals were monitored for signs of illness and weighed daily. Surviving mice were euthanized 28 days after infection, and serum was collected for serology. Research was approved and conducted in compliance with the guidelines of the NIAID/RML Institutional Animal Care and Use Committee (IACUC). The facility where this research was conducted is fully accredited by the Association for the Assessment and Accreditation of Laboratory Animal Care International (AAALAC) and has an approved Office of Laboratory Animal Welfare (OLAW) Assurance (#A4149-01). All procedures were conducted by trained personnel under the supervision of veterinarians, and all invasive clinical procedures were performed while animals were anesthetized. Early endpoint criteria, as specified by the IACUC approved scoring parameters, were used to determine when animals should be humanely euthanized.

### Statistical analysis

All data were analyzed using the GraphPad Prism v6.0 software. For the viral attachment, internalization, and membrane fusion experiments, a Student’s *t*-test was used to evaluate differences between 6D6 and control IgG. To assess the weight loss of mice, we performed a 2-way repeated-measures analysis of variance (ANOVA), followed by multiple *t*-tests comparing the average weights of 6D6-treated and untreated (control) mice at each time point, using the Holm–Sidak method. P values less than 0.05 were considered to be statistically significant.

## Additional Information

**How to cite this article**: Furuyama, W. *et al*. Discovery of an antibody for pan-ebolavirus therapy. *Sci. Rep.* **6**, 20514; doi: 10.1038/srep20514 (2016).

## Supplementary Material

Supplementary Information
