# Supplementary material for: Discovery of an antibody for pan-ebolavirus therapy
Source: Sci Rep. 2016 Feb 10;6:20514. doi: 10.1038/srep20514 (PMC4748290; doi:10.1038/srep20514)
Supplement: Supplementary Information [file srep20514-s1.pdf]

Supplementary Information

Discovery of an antibody for pan-ebolavirus therapy

Wakako Furuyama[1], Andrea Marzi[2], Asuka Nanbo[3], Elaine Haddock[2], Junki Maruyama[1], Hiroko Miyamoto[1], Manabu Igarashi[1], Reiko Yoshida[1], Osamu Noyori[1]†, Heinz Feldmann[2] & Ayato Takada[1].

[1]Division of Global Epidemiology, Research Center for Zoonosis Control, Hokkaido University, Sapporo, Japan
[2]Laboratory of Virology, Division of Intramural Research, National Institute of Allergy and Infectious Diseases, National Institutes of Health, Rocky Mountain Laboratories, Hamilton, Montana, USA
[3]Department of Cell Physiology, Graduate School of Medicine, Hokkaido University, Sapporo, Japan

†Current address
International Research Center for Medical Sciences, Kumamoto University, Kumamoto, Japan

Correspondence and requests for materials should be addressed to A.T. (atakada@czc.hokudai.ac.jp).

Supplementary Figures 1-2

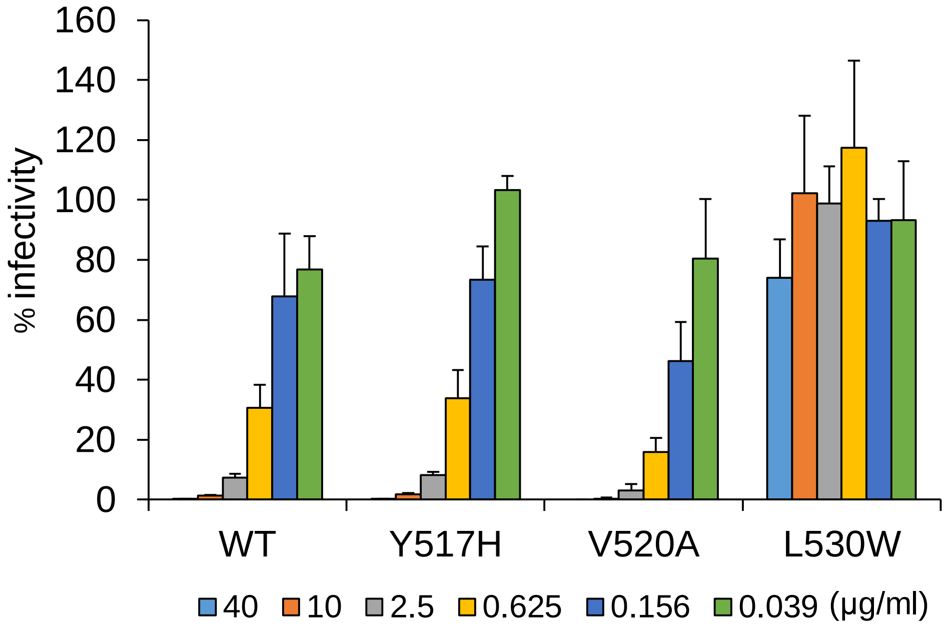

Supplementary Figure 1. Identification of the key amino acid residue on the escape mutant RESTV GP selected by MAb 6D6. Two RESTV GP mutants had the same Gly-to-Glu substitution at position 529, which is the corresponding position of the EBOV GP mutants, whereas three amino acid changes were found in the other 4 mutants of RESTV GP: Tyr at position 517, Val at position 522, and Leu at position 530 were replaced with His, Ala, and Trp, respectively (**Fig. 2a**). To clarify which amino acid change was critical for escaping from the 6D6 neutralization, we generated RESTV GP mutants with single amino acid substitutions for each position (Y517H, V520A, and L530W), and investigated the neutralizing activity of MAb 6D6 against VSV pseudotyped with these single amino acid mutants of RESTV GP.

a

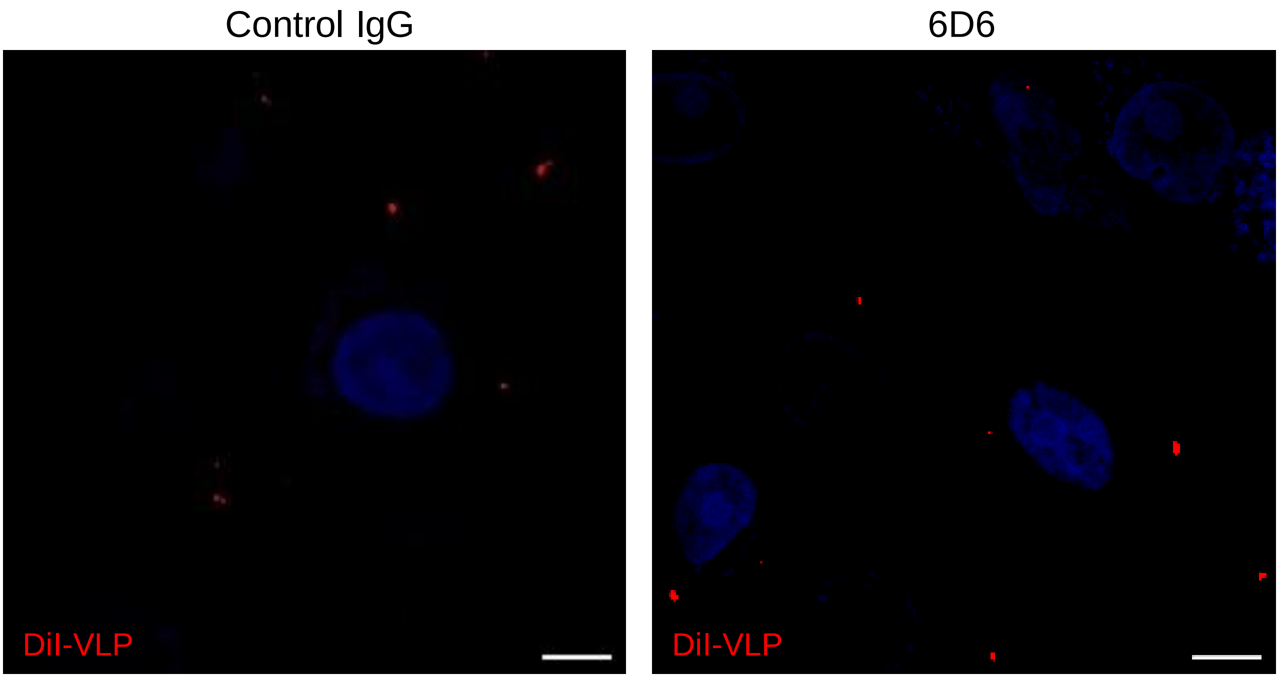

b

Control IgG 6D6

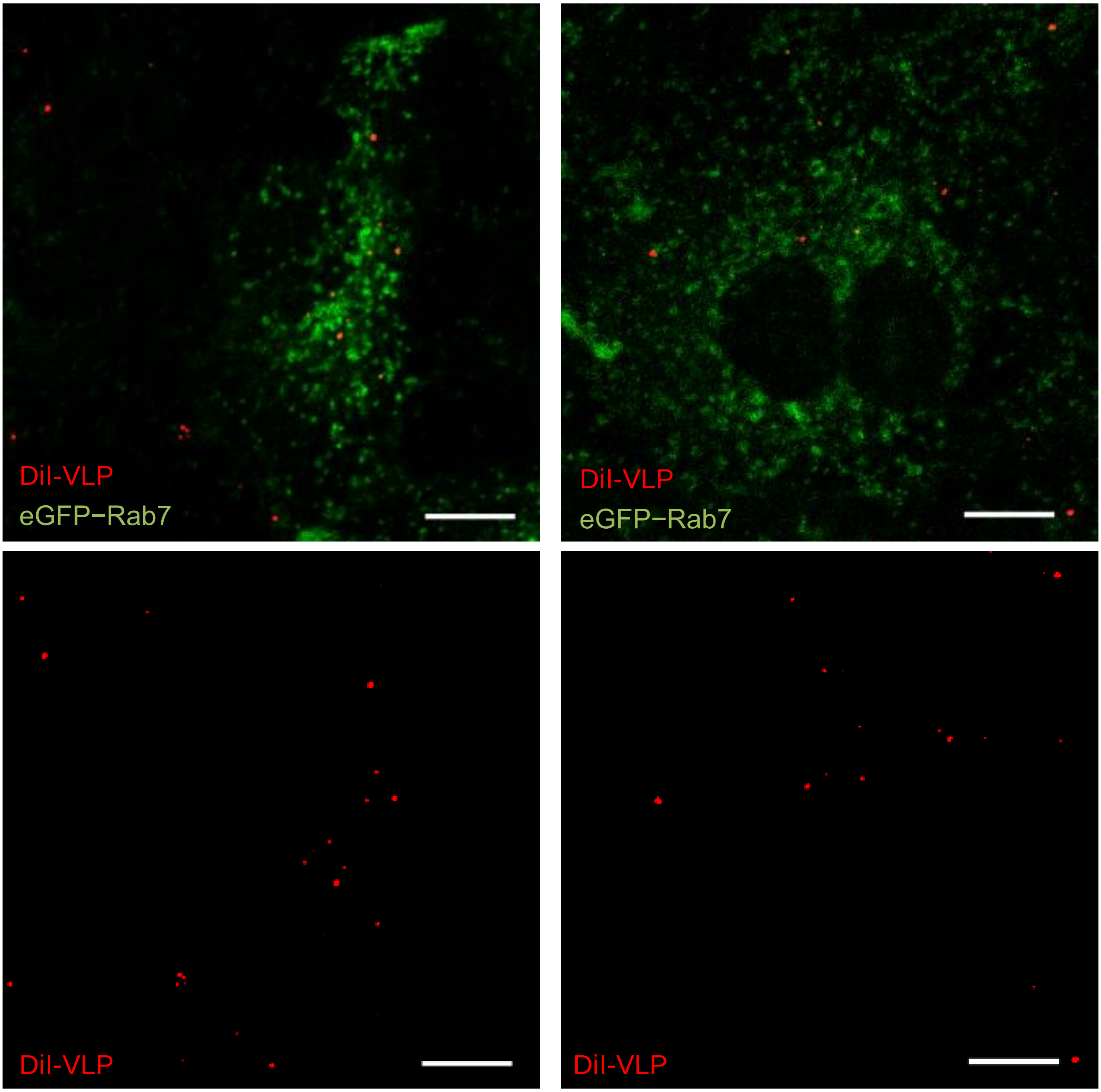

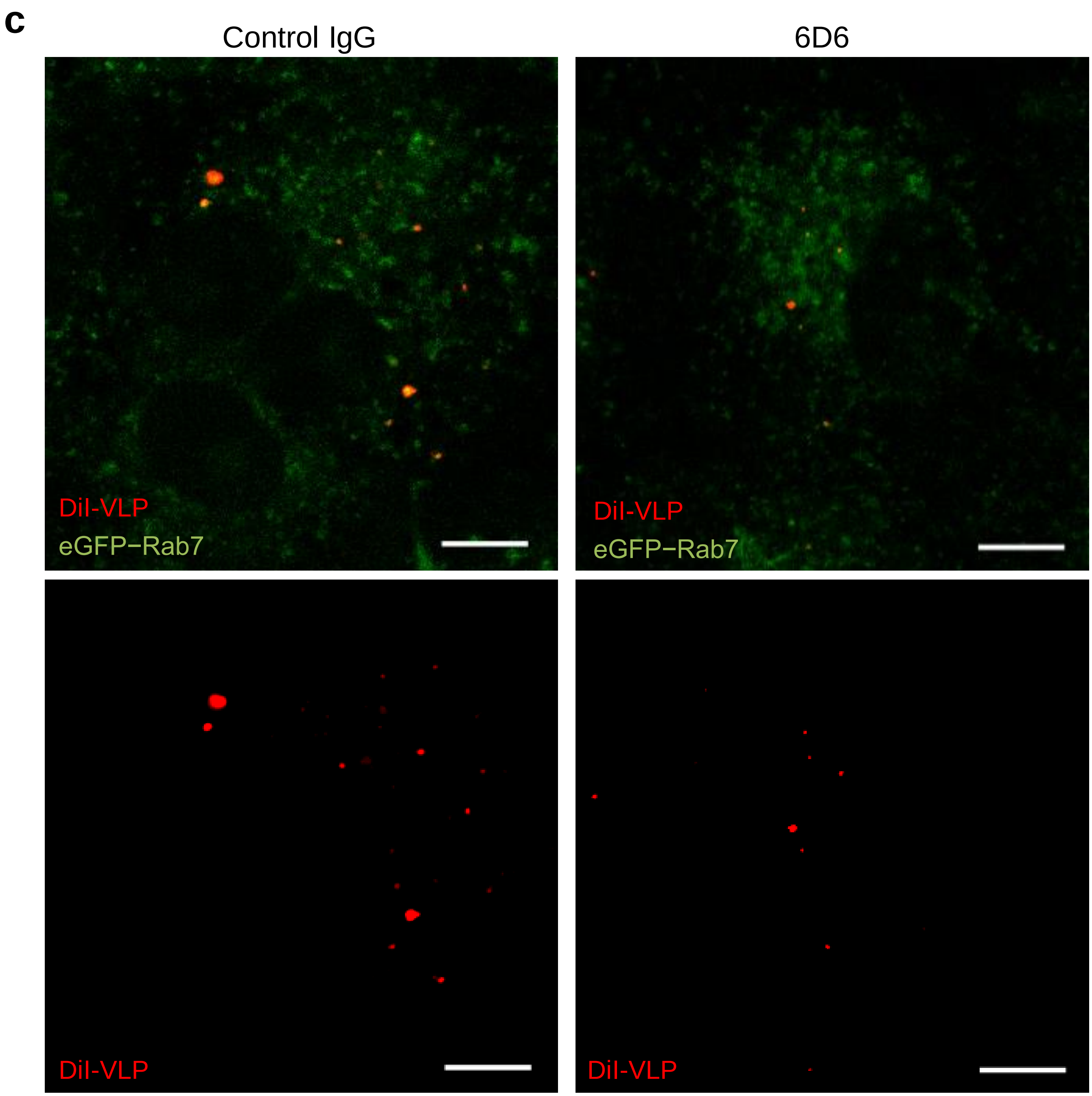

Supplementary Figure 2. Magnified images of DiI-labelled VLPs shown in Fig. 3. (**a**-**c**) Control IgG- and 6D6-treated DiI-labelled VLPs were inoculated into confluent Vero E6 cells expressing eGFP-Rab7 and incubated for 30 min on ice. After adsorption, the cells were incubated for 0 (**a**), 2 (**b**), and 6 h (**c**) at 37˚C. DiI signals on the cell surface (**a**) and in the cytoplasm (**b** and **c**) were monitored by confocal laser scanning microscopy. Scale bars represent 10 μm
